# Machine learning model comparison for freezing of gait prediction in advanced Parkinson’s disease

**DOI:** 10.3389/fnagi.2024.1431280

**Published:** 2024-06-25

**Authors:** Jeremy Watts, Martin Niethammer, Anahita Khojandi, Ritesh Ramdhani

**Affiliations:** ^1^Department of Mathematics, University of Tennessee, Knoxville, TN, United States; ^2^Zucker School of Medicine at Hofstra/Northwell, Hempstead, NY, United States; ^3^Center for Neurosciences, The Feinstein Institutes for Medical Research, Manhasset, NY, United States; ^4^Department of Industrial and Systems Engineering, University of Tennessee, Knoxville, Knoxville, TN, United States

**Keywords:** deep brain stimulation, Parkinson’s disease, machine learning (ML), gait kinematics, freezing of gait (FOG)

## Abstract

**Introduction:**

Freezing of gait (FOG) is a paroxysmal motor phenomenon that increases in prevalence as Parkinson’s disease (PD) progresses. It is associated with a reduced quality of life and an increased risk of falls in this population. Precision-based detection and classification of freezers are critical to developing tailored treatments rooted in kinematic assessments.

**Methods:**

This study analyzed instrumented stand-and-walk (SAW) trials from advanced PD patients with STN-DBS. Each patient performed two SAW trials in their OFF Medication—OFF DBS state. For each trial, gait summary statistics from wearable sensors were analyzed by machine learning classification algorithms. These algorithms include k-nearest neighbors, logistic regression, naïve Bayes, random forest, and support vector machines (SVM). Each of these models were selected for their high interpretability. Each algorithm was tasked with classifying patients whose SAW trials MDS-UPDRS FOG subscore was non-zero as assessed by a trained movement disorder specialist. These algorithms’ performance was evaluated using stratified five-fold cross-validation.

**Results:**

A total of 21 PD subjects were evaluated (average age 64.24 years, 16 males, mean disease duration of 14 years). Fourteen subjects had freezing of gait in the OFF MED/OFF DBS. All machine learning models achieved statistically similar predictive performance (*p* < 0.05) with high accuracy. Analysis of random forests’ feature estimation revealed the top-ten spatiotemporal predictive features utilized in the model: foot strike angle, coronal range of motion [trunk and lumbar], stride length, gait speed, lateral step variability, and toe-off angle.

**Conclusion:**

These results indicate that machine learning effectively classifies advanced PD patients as freezers or nonfreezers based on SAW trials in their non-medicated/non-stimulated condition. The machine learning models, specifically random forests, not only rely on but utilize salient spatial and temporal gait features for FOG classification.

## Introduction

Gait impairment is seen early in Parkinson’s disease (PD), with studies showing evidence of subtle changes in the prodromal stages (Mirelman et al., 2011; McDade et al., 2013). Measurement of gait can be grouped into the following strata: (1) spatiotemporal characteristics such as stride length, stance time, swing time, single limb support, and stride time that are expressed as means from multiple continuous steps; (2) dynamic features of spatiotemporal characteristics (gait variability or stride-to-stride variation) reflected as standard deviations or coefficient of variation derived from the same with-in subject steps; (Lord et al., 2013) and (3) joint kinematics. Characteristic PD gait changes include reduced stride lengths (Morris et al., 1996, 1998), decreased velocity, lack of arm swing, and multistep turns (Hausdorff, 2009; Galna et al., 2015). It also consists of freezing of gait (FOG), which manifests in three ways: inability to start walking or an arrest of forward progression; trembling of the legs in place; and moving forward with very small steps (Nutt et al., 2011). These episodes can be triggered by activities such as approaching a chair, passing through narrow passages, or during turns (Nutt et al., 2011). The prevalence of FOG ranges from 7.1% (Giladi et al., 2001) in early disease to 92% at more advanced stages (Virmani et al., 2015).

Paroxysmal FOG significantly increases fall risk and dramatically reduces quality of life. This phenomenon is often determined retrospectively through questionnaires on non-motor symptoms and gross motor disturbances, which have been predictive of the conversion of non-freezers to freezers (Banks et al., 2019; D’Cruz et al., 2020). Current clinimetric-based phenotypes of this heterogeneous condition are not prognosticative and provide limited insight into treatment response. The majority of freezing of gait studies utilize machine learning to detect and predict FOG episodes using wearable sensors [refer to review (Pardoel et al., 2019)] rather than classifying freezers from non-freezers (Mancini et al., 2023; Virmani et al., 2023). The latter provides the potential to stratify and even phenotype patients with considerable motoric heterogeneity. Along these lines, classifying patients with FOG who have chronic deep brain stimulation of the subthalamic nucleus (STN-DBS) offers an opportunity to probe FOG kinematic features in a dual-treated (medication/stimulation) cohort. Therefore, this study evaluated various machine learning (ML) modeling approaches to classify freezers from non-freezers following stimulation and medication washout of an advanced PD cohort with chronic STN-DBS.

## Materials and methods

### Patient cohort and gait assessment

The study procedures have been previously reported (Ramdhani et al., 2023). Twenty-one subjects met the inclusion criteria of having idiopathic PD with bilateral STN-DBS (greater than three months) and an underlying gait disorder defined as a score of 2 or 3 on the gait sub-score of the Movement Disorders Society (MDS)-Unified Parkinson’s Rating Scale Part III (UPDRS). Each patient performed two stand-and-walk (SAW) trials in their OFF Medication—OFF DBS state. The OFF medication state was achieved following an overnight (12 h) withdrawal of PD medications prior to the assessment, while the OFF DBS state consisted of a 50-min stimulation washout in the laboratory. Each SAW consisted of a 30-s standing period, followed by a 7 m walk, a 180-degree turn, and a return walk. For each trial, gait summary statistics from full-body (wrists, feet, sternum, and lumbar spine) Opal wearable sensors (Mobility Lab, APDM, Portland, OR, USA) were analyzed by ML classification algorithms. The measurements of interest assessed during the SAW included spatiotemporal features of gait and circumduction along with lumbar and trunk dynamics (Ramdhani et al., 2023). Walking aides were permitted for the study, and their influence was reduced in the models by excluding upper limb and postural sway parameters from the analysis. The Institutional Review Boards of Northwell Health and the University of Tennessee (UTK-IRB-19-05559-XP) approved all data collection and analysis.

### Machine learning

The algorithms evaluated in this study include k-nearest neighbors (KNN), logistic regression, naïve Bayes, random forest, and support vector machines (SVM). These models were selected due to their interpretability, robustness with input features, and success in binary classification tasks within the healthcare domain (Cortes and Vapnik, 1995; Breiman, 2001; Hand and Keming, 2007; Agresti, 2012; Tayeb et al., 2017). Each algorithm was tasked with identifying freezers, defined as patients whose SAW trials MDS-UPDRS FOG score was non-zero as assessed by a trained movement disorder specialist. Each algorithm’s performance was evaluated using stratified five-fold cross-validation, wherein the total cohort is separated into five portions such that iterative one portion is utilized as the testing set and the remaining four portions are used for training. Additionally, within each portion, samples are stratified such that the target variable is proportionally consistent with the population - thereby improving the generalizability of the models’ performance. Hyperparameter tuning was performed for each algorithm. Finally, feature ranking was conducted using the Gini index of node impurities to identify gait features prioritized by the random forest model (Tan et al., 2016). Each algorithm was implemented in Python using the Sklearn machine learning library (Pedregosa et al., 2011).

Predictive metrics, accuracy, sensitivity, specificity, positive predictive values (PPV), negative predictive values (NPV), F1 score, and the area under the receiver operating characteristic curve (ROC AUC) summarizing each ML algorithm’s performance were calculated following the analysis. Each algorithm’s performance metrics summarize the ratio of true positives (TP), true negatives (TF), false positives (FP), and false negatives (FN) as follows,

$$A\,c\,c\,u\,r\,a\,c\,y=\frac{T\,P+T\,N}{T\,P+T\,N+F\,P+F\,N}$$

$$S\,e\,n\,s\,i\,t\,i\,v\,i\,t\,y=\frac{T\,P}{T\,P+F\,N}$$

$$S\,p\,e\,c\,i\,f\,i\,c\,i\,t\,y=\frac{T\,N}{T\,N+F\,P}$$

$$P\,P\,V=\frac{T\,P}{T\,P+F\,P}$$

$$N\,P\,V=\frac{T\,N}{T\,N+F\,N}$$

$$F\,1=\frac{2\,T\,P}{2\,T\,P+F\,P+F\,N}$$

Additionally, each metric’s 95% confidence interval was determined to provide bounds to the predictive metrics.

## Results

The study cohort consisted of twenty-one chronic STN-DBS patients (16 males/5 females, with a mean disease duration of 14 [range 3–40 years], with an average age of 64 [range 39–80 years]). Mean gait subscore of the MDS-UPDRS subscale III was 2.4 (SD 0.9). Fourteen subjects were classified as freezers based on a score > 1 on the freezing of gait subscore in the OFF MED/OFF DBS state.

The ML classification models’ performance metrics (mean and 95% confidence interval) are presented over stratified five-fold cross-validation in Table 1. Overall, the models perform statistically similarly as assessed via *t*-test (*p* < 0.05) across all metrics and models. These results suggest that each model is equally capable of discriminating between freezers and non-freezers.

**TABLE 1:** ML algorithms’ performance metrics.

	K-nearest neighbors	Logistic regression	Naïve Bayes	Random forest	SVM
Accuracy	0.60 ± 0.35	0.69 ± 0.26	0.75 ± 0.24	0.76 ± 0.24	0.73 ± 0.24
Sensitivity	0.67 ± 0.35	0.63 ± 0.38	0.71 ± 0.24	0.79 ± 0.27	0.71 ± 0.24
Specificity	0.60 ± 0.52	0.85 ± 0.28	0.85 ± 0.28	0.70 ± 0.34	0.80 ± 0.56
PPV	0.72 ± 0.33	0.87 ± 0.23	0.85 ± 0.28	0.80 ± 0.27	0.90 ± 0.28
NPV	0.43 ± 0.40	0.62 ± 0.31	0.65 ± 0.26	0.73 ± 0.31	0.53 ± 0.45
F1 Score	0.66 ± 0.34	0.69 ± 0.31	0.77 ± 0.24	0.79 ± 0.24	0.77 ± 0.18
ROC AUC	0.64 ± 0.34	0.74 ± 0.21	0.78 ± 0.24	0.75 ± 0.23	0.76 ± 0.27

**FIGURE 1:**
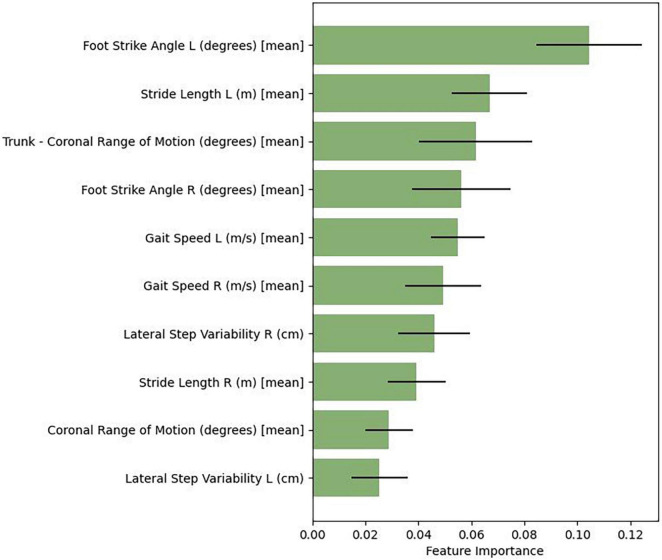
Gait feature importance derived from random forest.

Similar to the other algorithms assessed, random forest is capable of feature estimation, but accounts for nonlinear interactions between features. We therefore evaluated the specific weights or coefficients corresponding to the relative importance of each feature (gait summary statistic) to its classification prediction. Figure 1 presents the relative feature importance of the top-ten predictive features utilized in the random forest model. These features include both spatial (foot strike angle, coronal range of motion [trunk and lumbar], stride length, toe-off angle) and temporal (gait speed and lateral step variability) gait features.

## Discussion

This study shows the performance of ML algorithms in classifying freezers from non-freezers in an advanced PD cohort following a washout of medication and stimulation. The five evaluated ML models—logistic regression, KNN, naïve Bayes, random forest, and SVM—performed statistically similarly.

FOG detection algorithms have been explored to accurately detect freezing episodes based on data streams from inertial sensors; the classifiers shown to be most robust in FOG detection are convolutional neural networks, support vector machines, and decision trees (Pardoel et al., 2019; Borzi et al., 2021). Among several of the PD freezer/non-freezer classification studies (Park et al., 2021; Virmani et al., 2023), logistic regression and random forests classifiers were utilized and demonstrated good accuracy. Our study adds to the growing compendium of ML based freezing of gait classification studies in PD using gait kinematics. Each classification algorithm performed statistically similar in this study– highlighting each model’s ability to effectively use inertial sensor data and clinimetrics to differentiate freezers from non-freezers. Compared to the others, random forest is an ensemble method that accounts for interactions between features and can aid in determining which ones are most pertinent for prediction.

The random forest algorithm from this study highlighted key gait predictive features: foot strike angle, trunk and lumbar range of motion, stride length, gait speed, step variability, and toe-off angle. Further examination of these extracted gait features show they were salient to the phenotypic nuances that have been reported in those with freezing of gait. In a recent longitudinal study (Glover et al., 2020), investigators assessed spatiotemporal gait parameters of 26 freezers and 31 non-freezers over 12-months in the medicated state. They showed that freezers had a faster decline in mean stride length, stride velocity, swing (%), single support (%), and variability of single support compared to non-freezers. Plotnik et al. (2005) reported that foot swing time was more asymmetric and uncoordinated among freezers, while poor control of rhythmicity (measured by an increase in stride-to-stride variability) in the interictal period with stride length and foot strike (Hausdorff et al., 2003; Pillai et al., 2022) serve as a marker in those who experience freezing of gait as well as those with a history of falling (Schaafsma et al., 2003). Park et al. (2021) utilized a stepwise regression analysis based on 360-degree turning characteristics to determine six features to classify freezers from non-freezers: outer step length, hip ROM, ankle ROM, total distance of center of mass (COM), maximum anti-phase, and outer contralateral temporal coordination. In their analysis, random forest yielded the greatest accuracy of classifying those with FOG. With respect to PD subjects treated with DBS, O’Day et al. (2020) used a novel gait paradigm to demonstrate that freezers’ non-freezing gait is more arrhythmic than controls and correlates with the percent time of freezing. Additional work is needed to identify whether the random forest’s features reported in this study are also relevant in specific or underrepresented populations (i.e., gender, race, disease severity, etc.).

Biomechanical features of PD gait also differ based on the presence or absence of freezing. Non-freezers have reduced range of motion (ROM) of the knee joint (Albani et al., 2014) while reduced acceleration of the pelvis in the vertical and anteroposterior planes were displayed among freezers and those individuals who fall (Latt et al., 2009). Our model’s prioritization of lumbar and trunk ROM aligns with reports that pelvic rotation is necessary to modulate stride length, and as the disease advances, the rise in truncal rigidity leads to the inability of the pelvis to move out of phase with thoracic rotation to increase stride length and stride velocity (Albani et al., 2014). This ultimately causes a compensatory shift to the lower limb joints whereby ROM at the hips, knees, and ankles adjust to limited rotations of the spine (Huang et al., 2010).

As evidenced by our findings, the gait feature set demonstrates salient spatiotemporal elements that have previously been associated with freezers—reinforcing the robustness of these ML approaches regardless of the underlying PD treatments applied to the cohort. This has potential clinical bearing on preoperative DBS assessments that rely on the Core Assessment Program for Surgical Intervention Therapies (CAPSIT) testing. During those assessments, FOG may not be observed despite the patient’s historical accounts. Identifying a gait feature set associated with patients who have freezing episodes in the unmedicated state leverages ML and inertial sensors as viable compliments to characterizing gait severity for those individuals under consideration for an advanced therapy. As highlighted in this study, stimulation and medication washout are necessary to investigate the underlying gait disorder based on the greater prevalence of freezing in the unmedicated state. However, conducting this kind of clinical evaluation in the office environment can be time-consuming and arduous, thus underscoring the efficiency of gait kinematics plus ML to decipher and stratify complex motor symptoms into clinically meaningful disease-related characteristics.

## Conclusion

In summary, these results reveal statistically similar performances of machine learning algorithms in classifying advanced PD patients as freezers or non-freezers based on SAW trials in their medication and stimulation naïve state. Additional analysis on the random forest algorithm demonstrates its capability to extract salient spatial and temporal gait features in this classification.

## Data availability statement

The datasets presented in this article are not readily available because further inquiries including requests for datasets and raw data can be directed to the corresponding author. Requests to access the datasets should be directed to RR, rramdhani@northwell.edu.

## Ethics statement

The studies involving humans were approved by the Institutional Review Boards of Northwell Health and the University of Tennessee. The studies were conducted in accordance with the local legislation and institutional requirements. The participants provided their written informed consent to participate in this study.

## Author contributions

JW: Formal analysis, Writing – original draft, Writing – review & editing. MN: Project administration, Writing – review & editing. AK: Conceptualization, Formal analysis, Methodology, Supervision, Writing – review & editing. RR: Conceptualization, Investigation, Methodology, Project administration, Supervision, Writing – original draft, Writing – review & editing.
